# miRNA Signatures in Bronchopulmonary Dysplasia: Implications for Biomarkers, Pathogenesis, and Therapeutic Options

**DOI:** 10.31083/j.fbl2907271

**Published:** 2024-07-25

**Authors:** Hajime Maeda, Xiaoyun Li, Hayato Go, Phyllis A. Dennery, Hongwei Yao

**Affiliations:** 1 Department of Molecular Biology, Cellular Biology, and Biochemistry, Brown University, Providence, RI 02912, USA; 2 Department of Pediatrics, Fukushima Medical University School of Medicine, 960-1295 Fukushima, Japan; 3 Providence Veterans Affairs Medical Center, Providence, RI 02908, USA; 4 Department of Medicine, Warren Alpert School of Medicine of Brown University, Providence, RI 02903, USA; 5 College of Pharmacy, Jinan University, 510632 Guangzhou, Guangdong, China; 6 Department of Pediatrics, Warren Alpert School of Medicine of Brown University, Providence, RI 02903, USA

**Keywords:** bronchopulmonary dysplasia, microRNA, hyperoxic exposure, mechanical ventilation, therapeutics

## Abstract

Bronchopulmonary dysplasia (BPD) is a chronic lung disease in premature infants characterized by alveolar dysplasia, vascular simplification and dysmorphic vascular development. Supplemental oxygen and mechanical ventilation commonly used as life-saving measures in premature infants may cause BPD. microRNAs (miRNAs), a class of small, non-coding RNAs, regulate target gene expression mainly through post-transcriptional repression. miRNAs play important roles in modulating oxidative stress, proliferation, apoptosis, senescence, inflammatory responses, and angiogenesis. These cellular processes play pivotal roles in the pathogenesis of BPD. Accumulating evidence demonstrates that miRNAs are dysregulated in the lung of premature infants with BPD, and in animal models of this disease, suggesting contributing roles of dysregulated miRNAs in the development of BPD. Therefore, miRNAs are considered promising biomarker candidates and therapeutic agents for this disease. In this review, we discuss how dysregulated miRNAs and their modulation alter cellular processes involved in BPD. We then focus on therapeutic approaches targeting miRNAs for BPD. This review provides an overview of miRNAs as biomarkers, and highlights potential pathogenic roles, and therapeutic strategies for BPD using miRNAs.

## 1. Introduction

Bronchopulmonary dysplasia (BPD) is the most common chronic lung disease in premature infants. At present, a majority of preterm infants are able to survive due to advances in prenatal and neonatal care, including the use of antenatal corticosteroids, effective ventilatory support, and surfactant treatment [1,2]. However, BPD remains the most common complication associated with prematurity, and is increasing in prevalence, most likely due to the increased survival of extremely low gestational age newborns. The incidence of BPD varies widely among countries from 11.8% to 56% [3,4]. This disease increases the economic, psychological, and social burdens to families due to prolonged stays in intensive care units, a need for home oxygen therapy at discharge, and repeated hospital admissions due to pulmonary exacerbations [5]. For example, BPD costs an average of ~$377,871 per infant during the first year of life [5–7].

Most infants born before 28 weeks of gestational age require ventilatory assistance and/or supplemental oxygen. The persistent airway obstruction seen in BPD is due to airway inflammation resulting from mechanical ventilation and oxygen therapy [8]. The risk factors for BPD also includes surfactant deficiency, ventilation, and oxygen toxicity. The pathology of BPD is characterized by alveolar dysplasia, vascular simplification, and dysmorphic vascular development [9]. Currently, steroids, surfactant, caffeine, and vitamin A are used for the treatment of BPD [10]. Unfortunately, these therapies have minimally reduced the prevalence of BPD and associated lung injury [11,12]. BPD also increases the risk of pulmonary and cardiovascular sequelae as well as adverse neurodevelopmental outcome [13–15]. Hence, there is an urgent need to develop new therapies to prevent lung injury and associated comorbidities in BPD.

microRNAs (miRNAs) are a class of small, non-coding RNAs with an average 22 nucleotides in length. In 1993, the first miRNA, lin-4, was discovered in *C. elegans* [16]. In 2000, the second miRNA, let-7, was characterized, and this miRNA is conserved in many species [17,18]. In addition to biogenesis, miRNAs are controlled by different mechanisms at the transcriptional and epigenetic levels. They bind to the 3*′* untranslated region (UTR) of mRNAs which decreases mRNA translation either via mRNA strand degradation or sequestration. miRNAs have pleiotropic roles in modulating cell differentiation, development, proliferation, inflammatory response, and apoptosis [19]. Dysregulation of miRNAs has been identified in various diseases, including BPD [20–23]. Oxidative stress, inflammation, apoptosis, senescence, as well as abnormal proliferation and angiogenesis contribute to lung pathological changes in BPD. In this review, we discuss the current understanding of how miRNA dysregulation impacts the pathogenesis of BPD. We also discuss the role of dysregulated miRNAs during early life, evolving and established BPD, which will support the concept of miRNA serving as biomarkers of this disease. Furthermore, we provide an overview of which miRNAs modulate key biological processes observed in BPD. Finally, we discuss the inhibitors and mimics of miRNAs that could serve as potential preventive or therapeutic agents for BPD.

## 2. Dysregulated miRNAs in Clinical Samples of Human BPD

miRNAs are abundant in tissues and fluids, such as blood and tracheal aspirates. These samples have been employed to evaluate the dysregulation of miRNAs in premature infants with BPD. Tables 1,2 (Ref. [19,24–59]) summarize upregulated and downregulated miRNAs in clinical samples from infants with BPD.

### 2.1 Blood

Accumulating evidence demonstrates that the expression of miRNAs is altered in the blood of premature infants with BPD. For example, expression of miR-203a (n = 4), miR-221 (n = 38) and miR-223 (n = 38) was significantly upregulated in the blood of patients with BPD at 28 days to 3 months of age compared to age-matched controls (n = 4 or n = 21) [31,32]. Since BPD is established at this age, these dyregulated miRNAs could be diagnostic biomarkers of this disease. In contrast, levels of miR-150 (at 28 days to 3 months of age), miR-574 (at *<*32 weeks gestational age), miR-206 (at 0–10 days of life from preterm infants born at *<*32 weeks gestation), and miR-29b (at 3–5 days of life in preterm infants born at *<*32 weeks gestation) were significantly decreased in the plasma of patients with BPD compared to controls (20 infants with BPD vs 10 non-BPD matched controls) [32,50,52,60]. In very low birth weight premature infants who developed BPD, blood levels of miR-133b and miR-7 were increased, whereas miR-152 and miR-30a-3p were reduced at 36 weeks postmenstrual age and the first two weeks of life (n = 15) compared to infants without BPD (n = 15) [24]. Blood levels of miR-17 were lowest in the first-week of life in infants who developed severe BPD (n = 5) compared to infants diagnosed with mild or moderate BPD (n = 20) [48], suggesting a correlation between miR-17 blood levels and disease severity. Additionally, these dysregulated miRNAs are altered in early life, which suggests that they could represent risk factors for developing BPD or serve as early biomarkers of this disease.

Blood exosomes are extracellular vesicles secreted by living cells into the circulating blood. Using next-generation sequencing and bioinformatic analysis, 328 miRNAs were upregulated and 90 miRNAs were downregulated in exosomes from umbilical cord vein blood of preterm infants who subsequently developed BPD (12 BPD infants vs 14 non-BPD infants). These miRNAs were those primarily enriched in the PI3K/Akt and angiogenesis-related signaling pathways [53]. Among them, blood levels of miR-200a-3p were increased whereas the expression of miR-103a and miR-185 was most significantly reduced [53]. Levels of miR-21 in serum extracellular vesicles on the 28th day of life were significantly increased in premature infants with severe BPD (n = 2) compared to those without BPD (n = 3) [28]. These findings suggest that alterations of miRNA in exosomes could serve as potential biomarkers of BPD. Further investigation is warranted to determine the mechanisms by which BPD alters these miR-NAs or vice versa, and to evaluate whether these changes in the blood correlate with their levels in the lung.

### 2.2 Tracheal Aspirates and Lung Tissues

In tracheal aspirates, levels of miR-628, miR-185, miR-545, and miR-378, detected by miRNA array, were significantly increased in premature infants with severe BPD (n = 17) compared to those with mild/moderate BPD (n = 8) [29]. This is in contrast to reduced expression of miR-185 in the blood of infants with BPD [53]. Expression of miR-1252, miR-1254, miR-130a, miR-20a, miR-20b, miR-378b, and miR-876 was higher in the tracheal aspirates from patients with severe BPD (n = 25, 23–28 weeks gestation) compared to gestational age-matched full-term controls (n = 25) [26]. Tracheal aspirates collected in the first postnatal week from premature infants who developed BPD exhibited significantly increased miR-34a (n = 5) and miR-199a (n = 10) expression compared to controls [25,27]. Also, miR-219 expression was markedly increased in tracheal aspirates and lung tissues of infants with severe BPD compared to post-conception age matched full-term infants (n = 30) [30]. In contrast, 28 miRNA levels were significantly decreased in the tracheal aspirates from premature infants with severe BPD compared to those with mild/moderate BPD [26,29]. Expression of miR-342 (n = 10), miR-30a (n = 9), miR-489 (n = 4), and miR-29b (n = 4) was decreased in tracheal aspirate cell pellets from neonatal infants with BPD (n = 10) [49,51,54]. Among these miR-NAs, miR-34a has been widely studied and is involved in Wnt signaling, TGF-*β* signaling, cell death, apoptosis, dysregulated vascularization, and abnormal cell proliferation [61]. These small clinical studies suggest that altered miR-NAs could serve as predictors of BPD or clinical biomarkers for the diagnosis of this disease. Larger clinical studies using blood, tracheal aspirates and lung tissues from premature infants in early life, before and after the establishment of BPD are warranted to validate these findings.

### 2.3 Pathways Associated with Dysregulated miRNAs

Pathway analysis indicated that differentially expressed miRNAs observed in BPD are associated with molecular and cellular functions including cell signaling, DNA replication, cell cycle, cell apoptosis, and inflammatory responses [25,29]. Further studies are warranted to better understand the role of specific miRNAs in altered cellular functions seen in BPD. Tracheal aspirates contain immune cells, epithelial cells, and mesenchymal stromal cells among others. Defining which cells in the tracheal aspirates show specific alterations in miRNAs that may contribute to BPD will be important.

## 3. Dysregulated miRNAs in Animal Models of BPD

### 3.1 miRNAs in Hyperoxia-Induced Animal Models of BPD

Animal models can help us better understand the role of miRNAs in the pathogenesis and potential treatment of BPD. Hyperoxia-exposed neonatal rodents are common animal models used to mimic BPD because hyperoxia alone results in lung injury similar to that seen in BPD. Other rodent models have used an inflammatory injuryto mimic BPD. We summarize the upregulation or downregulation of miRNAs in animal models of BPD (Tables 1,2). It is important to note that miRNA expression is altered differently depending on oxygen concentration, exposure time, animal species and cell types.

#### 3.1.1 Upregulated miRNAs Observed in Animal Models of BPD

Neonatal hyperoxia (60% oxygen for 21 days) resulted in the upregulation of various miRNAs at pnd2, pnd7 and pnd21 in mouse lung tissues [34], suggesting dynamic changes of these miRNAs. This is corroborated by the findings in a rat model showing that neonatal hyperoxia (60–85% oxygen for 14 days) upregulates miR-490 and miR-1193 at pnd3, miR-3584 at pnd7, and 19 miRNAs including miR-365 at pnd14 [36]. Lung miRNA expression profiling in neonatal mice exposed to hyperoxia (80% oxygen) or normoxia for either 14 days or 29 days showed several dynamically regulated miRNAs [33]. These miR-NAs include miR-411, miR-431, miR-699, miR-29a, and miR-29c. In neonatal rats exposed to hyperoxia (80 *±* 5% O_2_), miR-125b expression was significantly increased on pnd1, pnd4, and pnd7, and significantly decreased on pnd10 and pnd14 compared to air controls [45]. Expression of miR-154 increased steadily during development (from E10.5 to pnd2) and progressively disappeared from both the alveolar and bronchiolar compartments in the lungs of normoxia-exposed neonatal mice at pnd8, while the expression of miR-154 was maintained in these two compartments in hyperoxia (85% O_2_ from pnd0 to pnd8)-exposed lungs [44]. Expression of the miR-17~92 cluster in the lung of hyperoxia-exposed mice (85% O_2_ from pnd1 to pnd14) was lower than that in control mice [56]. Level of miR-421 was markedly upregulated in the lung of hyperoxia-exposed mice (85% O_2_ for 7 days) compared to air-exposed controls [46]. Neonatal hyperoxia (85% O_2_) also increased the expression of miR-219 and miR-203a in the lungs of both mice and rats [30,31]. Lung miR-29a expression was increased in mice exposed to hyperoxia as neonates (*>*90% oxygen for 3 days) followed by air recovery [40,42]. Exposure to hyperoxia (*>*90% oxygen) significantly increased the levels of lung miR-421 and miR-34a in neonatal mice [25,41]. Expression of miR-194 was increased in hyperoxia-exposed mice (*>*90% O_2_ for 3 days, and then recovered at room air for 10 days) and progressively increased during hyperoxic exposure [47]. miR-21 is found in the lungs and serum extracellular vesicles of hyperoxia-exposed neonatal mice (95% oxygen for 7 days) [28]. Using a rat model exposed to 95% oxygen for 10 days from pnd3 to pnd13, miR-141, miR-21, and miR-34a were significantly upregulated compared to controls [35,39]. In the lung of neonatal mice exposed to hyperoxia (95% oxygen for 3 days), miR-34a levels were increased at pnd3, pnd5, and pnd14 [39]. Further studies are warranted to determine whether these changes are common to both rats and mice exposed to hyperoxia as neonates and their significance in the development of lung injury.

#### 3.1.2 Downregulated miRNAs in Animal Models of BPD

Lung levels of miR-206 were significantly reduced in mice exposed to hyperoxia (60% oxygen exposure on pnd2, pnd7, pnd21) compared to air-exposed controls [60]. In newborn mouse lungs, miR-342 was significantly downregulated after 21 days of hyperoxic exposure (60% oxygen) compared to room air controls [55]. Interestingly, reduction of miR-299, miR-139p, miR-300, and miR-122 was observed at pnd2, and miR-335p and miR-714 at pnd7, and miR-720 at pnd21 in rodent lungs [34]. These findings suggest that neonatal hyperoxia decreases the expression of certain miRNAs in a dose- and time-dependent manner. This is confirmed by another study using a rat model showing that neonatal hyperoxia (60–85% oxygen for 14 days) reduced miR-377 at pnd3, downregulated 11 miRNAs including miR-139, miR-208a, and miR-188 at pnd7, and 16 miRNAs at pnd14 [36], illustrating the dynamic temporal expression of miRNAs in the lung after neonatal hyperoxia.

Using miRNA expression profiling, Dong *et al*. [33] identified 4 dynamically regulated miRNAs in the lungs of neonatal mice exposed to hyperoxia (80% oxygen) or room air for either 14 or 29 days. Ruiz-Camp *et al*. [39] reported that 14 miRNAs, including miR-29c and miR-34a, were dysregulated at pnd5 and pnd14 in neonatal mice exposed to hyperoxia (85% oxygen). In a neonatal hyperoxia exposure model (85% oxygen from 4 to 14 days of age), lung miR-489 expression was reduced. This may serve as a compensatory mechanism, because inhibiting miR-489 improved lung development after hyperoxia whereas miR-489 overexpression inhibited lung development [49]. Levels of miR-876–3p were decreased in the bronchoalveolar lavage fluid of mice exposed to hyperoxia (85% oxygen) from pnd3 to pnd14 [26]. Mu *et al*. [57] found that miR-20b was downregulated in the lungs of rats exposed to hyperoxia (95% oxygen) for 48 h. Wu *et al*. [59] demonstrated that miR-425 was downregulated in the lungs of rats with hyperoxia-induced lung injury (90% oxygen for 7 days). Zhang *et al*. [58] demonstrated that miR-214 expression was lower on pnd3, pnd7, and pnd14 in lungs of neonatal rats after 95% oxygen exposure compared to those from air exposed (21% oxygen) controls. Exposure to 95% oxygen for 10 days (from pnd3 to pnd13) in newborn rats downregulated the levels of 5 miRNAs, such as miR-342, in the lung at pnd13 [35]. Levels of miR-363 and miR-196a were downregulated in the lungs of neonatal mice exposed to hyperoxia (95% oxygen for 3 days) as demonstrated by miRNA arrays [37]. Neonatal hyperoxia (100% oxygen) from pnd1 to pnd4 significantly reduced lung miR-342 expression with a nadir at pnd2, pnd4, pnd7 and recovery at pnd14 [54].

Pathway analysis reveals that downregulated miRNAs are mainly related to immune and inflammatory processes, whereas upregulated miRNAs are associated with extracellular matrix remodeling. Different oxygen levels (60%–100%) and exposure durations (3–14 days) have been used to induce lung injury in rodents. Different durations of air recovery are also commonly used to investigate the long-term effects of neonatal hyperoxia on lung injury. Therefore, further studies are required to investigate the impact of different concentrations of oxygen, different durations of exposure and of air recovery on dysregulation of miRNAs. Additionally, further investigations are warranted to determine cell-specific changes in lung miRNAs after neonatal hyperoxia.

### 3.2 Dysregulated miRNAs in Intrauterine Infection/Inflammation Models

In the pups of pregnant rats endocervically inoculated with an *E. coli* suspension, levels of lung miR-184, miR-347, miR-181a, miR-204, miR-132, and miR-328b were upregulated, whereas expression of lung miR-122, miR-490 and another 8 miRNAs was downregulated after intrauterine infection compared to controls at pnd1 [38]. At pnd3, lung levels of miR-3559 were upregulated, whereas lung levels of miR-122, miR-490 and another 10 miR-NAs were downregulated in this model. Furthermore, at pnd14, lung miR-466b levels were upregulated, while expression of lung miR-122 was most significantly downregulated after intrauterine infection [38]. This is in corroboration with the findings that lung miR-122 was reduced in mice exposed to hyperoxia as neonates [34]. These data suggest that specific miRNAs dynamically participate in the progression of lung injury after intrauterine infection/inflammation, resulting in BPD.

## 4. Dysregulated miRNAs in Hyperoxia-Exposed Cultured Cells

Hyperoxia-exposed cells are commonly used to study mechanisms underlying the pathogenesis of BPD. We summarize upregulation or downregulation of miRNAs in cultured cells exposed to hyperoxia (Tables 1,2). The lung contains more than 40 types of cells. Thus, the impact of hyperoxia on miRNA expression in various lung cell types, such as alveolar epithelial, endothelial, and fibroblast cells, differs. In lung epithelial cells, expression of miR-219, and miR-421 was increased with exposure to hyperoxia (85% oxygen) for 6 h to 24 h [30,46]. Gilfillan *et al*. [43] demonstrated that miR-451 expression was significantly increased in murine lung endothelial cells exposed to 100% O_2_ for 16 h. In lung fibroblasts, levels of miR-219 and of the miR-34 family were increased with hyperoxic exposure (85% oxygen for 24 h) [30,39].

## 5. Consistently Dysregulated miRNAs in Various Models of BPD

Despite the vast heterogeneity of miRNAs that are altered in different animal models and in premature infants with BPD, some are more commonly upregulated. These include miRNA-34a, miR-219 and miR-421. Among them, miR-34a has been the most thoroughly studied. It is upregulated in the tracheal aspirates of premature infants with BPD and in animal models of this disease. This miRNA targets many genes, including *Wnt1*, *Snail*, *cdk4*, *SIRT1*, *Dll-4*, and modulates multiple pathways, such as Wnt, TGF-*β*, Notch and mTOR signaling [61]. As to downregulated miRNAs, miR-29b has been most widely studied. It is downregulated in cell pellets from tracheal aspirates and blood from premature infants with BPD and in lung tissues of rats after intrauterine infection/inflammation [50]. miR-29b plays an important role in modulating NF-kB, AKT and STAT3 signaling, which are associated with lung development [62,63]. Thus, reduction of miR-29 may disrupt lung development and cause the alveolar and vascular simplification seen in BPD.

## 6. Differentially Dysregulated miRNAs in Various Models of BPD

Several miRNAs, including miR-133, miR-20b and miR-185, are differentially altered between human and animal samples in BPD. For example, miR-133b expression was upregulated in blood collected during the first 2 weeks of life in 15 subjects with BPD compared to 15 sex-matched control subjects without BPD [33]. In contrast, lung miR-133b was downregulated in a rat model of BPD induced by intrauterine infection/inflammation at pnd3 [56]. Further study is warranted to determine whether miR-133b is secreted from lung tissues through exocytosis and transported into blood during the development of BPD. Compared to gestational age-matched full-term controls, miR-20a expression was increased in the tracheal aspirates of patients with severe BPD [26]. However, miR-20b expression was downregulated in the lungs of rats exposed to hyperoxia as neonates [57]. These discrepancies may be due to altered expression of miR-20b in different lung cells during the development of BPD. Similarly, miR-185 expression was significantly increased in the tracheal aspirates of premature infants with severe BPD compared to those with mild/moderate BPD [29]. In contrast, miR-185 was reduced in the blood of infants who develop BPD compared to controls who do not [53]. Whether miR-185 is reduced in endothelial cells and increased in lung epithelial cells during lung injury observed in BPD remains to be determined [64].

## 7. Impact of Dysregulation of miRNAs on Cellular Processes Involved in BPD

**Fig. 1.**
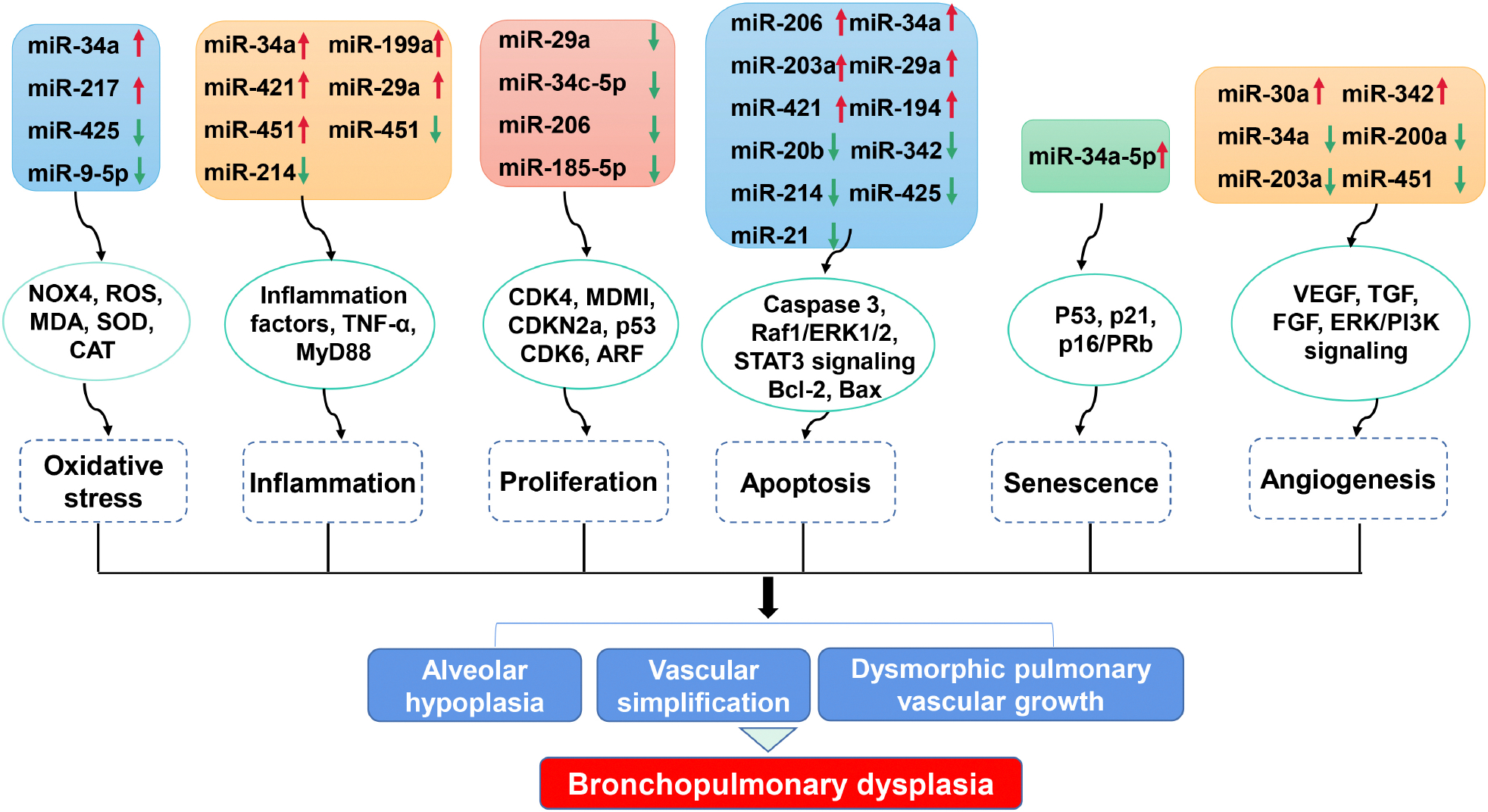
Impact of miRNA dysregulation on cellular processes involved in BPD. Dysregulated miRNAs modulate numerous cellular processes, including oxidative stress, inflammation response, proliferation, apoptosis, senescence and angiogenesis, via various targeted genes. These cellular processes participate in alveolar hypoplasia, vascular simplification and dysmorphic vascular growth, which are key pathological features of BPD. NOX4, NADPH oxidase 4; ROS, Reactive oxygen species; MDA, Malondialdehyde; SOD, Superoxide dismutase; CAT, Catalase; TNF, Tumor necrosis factor; CDK, Cyclin-dependent kinase; MDM1, Mouse double-minute 1; ARF, ADP-ribosylation factor; Raf1, Raf-1 proto-oncogene, serine/threonine kinase; ERK1/2, Extracellular signal-regulated protein kinase 1/2; STAT3, Signal transducer and activator of transcription 3; Bcl-2, B-cell lymphoma 2; VEGF, Vascular endothelial growth factor; TGF, Transforming growth factor; FGF, Fibroblast growth factor; ERK/PI3K, extracellular signal-regulated protein kinase/phosphoinositide 3-kinase.

Bioinformatic analyses have identified miRNAs as direct targets of specific cellular processes. Here we summarize the link between dysregulated miRNAs and cellular processes involved in BPD (Fig. 1, Table 3 (Ref. [19,25–27,31,40–43,46,47,51,53,54,57–60,65,66])).

### 7.1 Dysregulated miRNAs and Oxidative Stress

Expression of miRNAs can be altered by stresses such as exposure to hypoxia. Changes in miRNA expression under oxidative stress could regulate enzymes involved in miRNAs processing. Hypoxia inhibits the expression of DROSHA and DICER1, which could result in incomplete miRNA biogenesis [67]. Oxidative stress and radiation-induced DNA damage can activate p53 which affects the expression of several miRNAs [68]. Furthermore, miR-NAs regulate the expression of redox markers and antioxidants, including Cu/Zn SOD, catalase and glutathione peroxidase. Syed *et al*. [25] showed that hyperoxia-exposed mice have increased myeloperoxidase activity, which was significantly decreased in the lungs of global and epithelial cell-specific miR-34a knockout mice. Hyperoxic exposure increased miR-185 expression in cultured lung epithelial cells, which promoted DNA damage [69]. Therefore, there is a vicious cycle between dysregulated miRNAs and oxidative stress, which could further drive the progression of BPD.

### 7.2 Dysregulated miRNAs and Inflammatory Responses

Hyperoxia-exposed wild type mice had an increase in lung neutrophil infiltration, which was significantly decreased in the lung of global and type II epithelial cell-specific miR-34a knockout mice [25]. Inhibiting miR-199a expression attenuated hyperoxia-induced inflammatory responses including increased interleukin-6 (IL-6), tumor necrosis factor-*α* (TNF-*α*), and toll-like receptor 4 (TLR4), in the lung as well as impaired alveolarization and vascular function [27]. Inhibition of miR-421 decreased the levels of inflammatory factors (IL-6 and IL-1*β*) in neonatal hyperoxia-exposed mice by targeting fibroblast growth factor 10 (Fgf10), thereby alleviating pathological alterations [41]. By targeting miR-421, the long non-coding RNAs (lncRNA) imprinted and accumulated in the nucleus (Rian) attenuated hyperoxia-induced lung injury via inhibition of the inflammatory response [46]. Overexpression of the lncRNA for taurine upregulated gene 1 (TUG1) inhibited the production of IL-6 and IL-1*β*. These inhibitory effects of TUG1 were reversed by overexpression of miR-29a in MLE12 cells exposed to hyperoxia [42]. Altogether, these miRNAs promote lung inflammatory responses in neonatal hyperoxia. In contrast, treatment with an miR-451 inhibitor in both air and neonatal hyperoxia-exposed mice resulted in increased expression of the pro-inflammatory cytokines IL-6 and IL-1*β* [43], suggesting that this miRNA exerts inhibitory effects on lung inflammation in BPD.

### 7.3 miRNAs and Cell Proliferation

GRB2-associated-binding protein 1 (*GAB1*) is a target gene of miR-29a. Inhibition of miR-29a promoted proliferation of MLE12 cells exposed to hyperoxia, and also protected against neonatal hyperoxia-induced lung injury through GAB1 upregulation [40]. Extracellular vesicle miR-34c-5p derived from bone mesenchymal stem cells enhanced proliferation and migration in human pulmonary microvascular endothelial cells, and inhibited hyperoxic lung injury [70]. Overexpression of miR-103a-3p and miR-185–5p significantly enhanced the proliferation and migration of normal human umbilical vein endothelial cells, whereas overexpressing miR-200a-3p inhibited these responses [53]. A better understanding of the roles of these dysregulated miRNAs in modulating lung injury observed in BPD is needed.

### 7.4 miRNAs and Apoptosis

Certain miRNAs directly target key molecules involved in apoptotic pathways, including caspase and Bcl-2 family members [71]. For instance, miR-34a, miR-203a, miR-421, miR-29a, and miR-194 are pro-apoptotic, whereas miR-342, miR-214, miR-20b, and miR-425 inhibit apoptosis in cultured lung epithelial cells and mouse lungs exposed to hyperoxia. Transfection of miR-34a mimics further increased hyperoxia-induced apoptosis in type II alveolar epithelial cells, and these effects were decreased by an miR-34a inhibitor or genetic disruption [25]. In RLE-6TN cells, miR-203a transfection caused apoptosis [31]. Inhibition of miR-29a and miR-421 reduced neonatal hyperoxia-induced apoptosis in the lung by upregulating GAB1 and Fgf10, respectively. Consequently, inhibition of these miRNAs protected against neonatal hyperoxia-induced lung injury in mice [40–42,46]. Overexpression of lncRNA CASC2 inhibited hyperoxia-induced apoptosis in pulmonary bronchial epithelial cells and lung injury by inhibiting miR-194 [47]. Upregulating miR-194 blocked these effects.

In contrast, miR-342 overexpression decreased type II alveolar epithelial cell apoptosis under hyperoxic conditions. This was associated with inhibition of Spred3, and the pro-survival Raf1/ERK1/2 signaling pathway [54]. Overexpression of miR-214 inhibited apoptosis in rat bronchial embryonic lung epithelial cells by downregulating the STAT3 pathway, and subsequently protected against neonatal hyperoxia-induced lung injury in rodents [58]. miR-20b overexpression attenuated hyperoxia-induced mitochondrial dysfunction-mediated apoptosis by targeting Mfn1 and Mfn2 [57]. This also inhibited hyperoxia-induced acute lung injury in adult mice. Therefore, miR-NAs play a significant role in regulating apoptosis during hyperoxic lung injury.

### 7.5 miRNAs and Senescence

Cellular senescence refers to the irreversible arrest of cell proliferation, which is characterized by a senescence-associated secretory phenotype and resistance to apoptosis. We reported that early programmed senescence orchestrates postnatal lung development whereas later hyperoxia-induced senescence causes lung injury [72]. miRNAs play a role in modulating senescence by potentially targeting genes on the p53, p21 and p16/pRb pathways. Furthermore, miRNAs also can regulate the actin cytoskeleton structure that contributes to the enlarged and flattened cell morphology, a characteristics of the senescence phenotype [73]. We reported that miR-34a mediates hyperoxia-induced senescence in cultured lung epithelial cells by upregulating the KLF4/p21 signaling pathway [66]. Expression of miR-34a-5p was increased in cultured lung epithelial cells and in the lungs of newborn mice exposed to hyperoxia, as well as in the lung of premature infants requiring mechanical ventilation. Further studies are warranted to understand the contribution of miR-34a to lung injury in BPD using larger animal models.

### 7.6 miRNAs and Angiogenesis

Aberrant angiogenesis is a key feature of the lung injury observed in BPD. Treatment with an miR-34a inhibitor protected against neonatal hyperoxia-induced vascular simplification in mice, and this was partially mediated via the Ang1/Tie signaling pathway [25]. Inhibiting miR-451 improved pulmonary vascular growth and alveolar simplification in neonatal hyperoxia-exposed mice. This was associated with sustained expression of macrophage migration inhibitory factor and increased expression of vascular endothelial growth factor A (VEGFA), Ang1, Ang2, and the Ang receptor Tie2 [43]. Knockdown of miR-203a protected against neonatal hyperoxia-induced alveolar simplification in rats by increasing VEGFA expression [31]. In contrast, overexpression of miR-342 increased pulmonary vessel density in neonatal mice exposed to hyperoxia [54]. The miR-30a mimic increased angiogenic sprouting in cultured human pulmonary microvascular endothelial cells by inhibiting delta-like ligand 4 (Dll4). Interestingly, these effects were observed in female but not male cells. Deletion of miR-30a expression eliminated the female resilience to neonatal hyperoxic lung injury, suggesting important roles of miRNAs in driving the sexual dimorphism observed in BPD [51,74]. Overall, these findings suggest that miRNAs could be therapeutic targets to prevent lung injury seen in BPD through modulation of angiogenesis.

## 8. Modulators of miRNAs Used as Therapeutic Agents for BPD

**Fig. 2.**
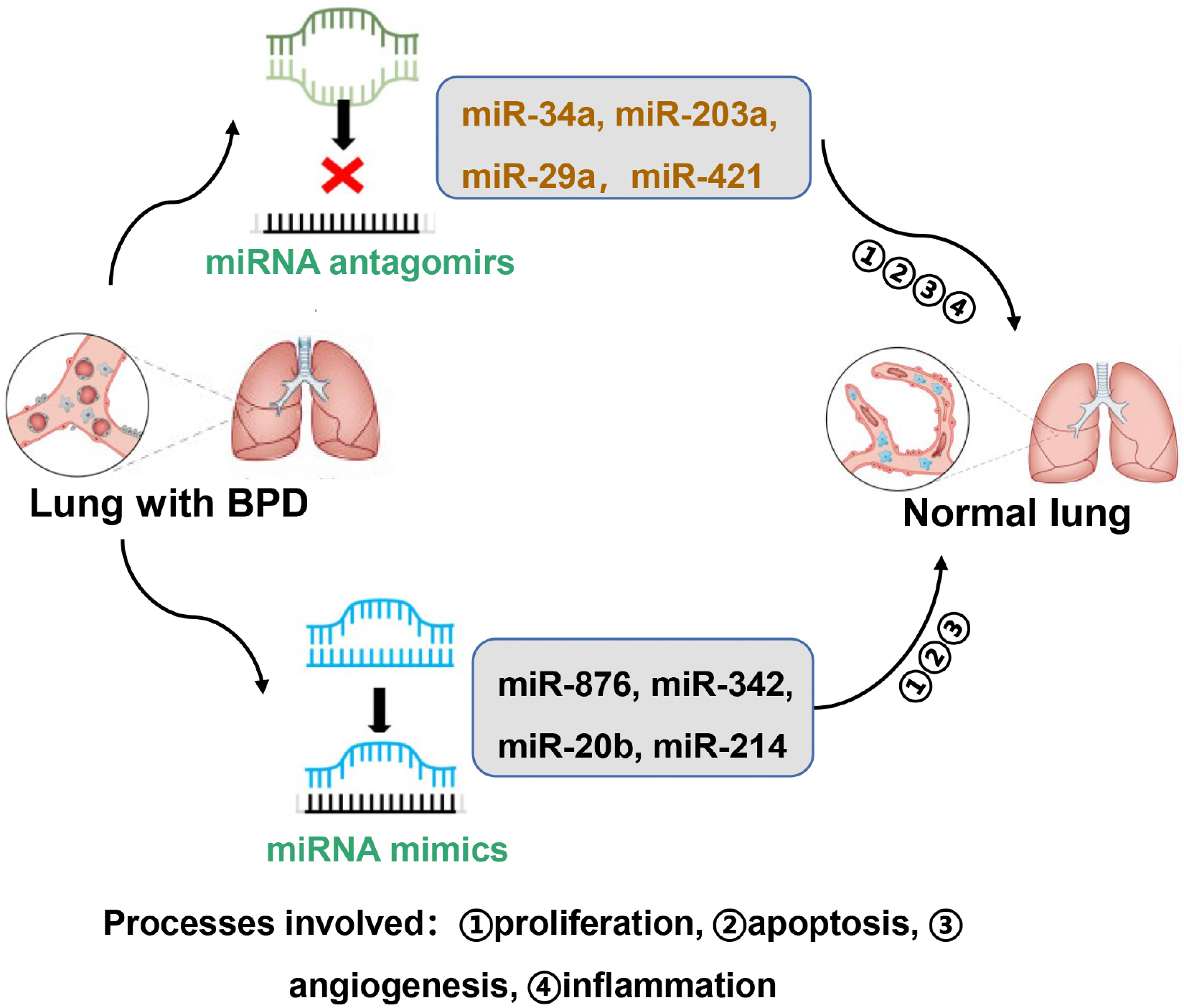
Modulators of miRNAs as potential therapeutic agents to treat BPD. Certain miRNA inhibitors and mimics have been used in animal models of BPD. These include miRNA antagomirs of miR-34a, miR-203a, miR-29a, and miR-421, as well as miRNA mimics of miR-876, miR342, miR-20b, and miR-214. These miRNAs impact alveolar and vascular simplification by modulating proliferation, apoptosis, inflammation and/or angiogenesis.

Although many miRNAs are used as biomarkers in clinical medicine and as potential therapeutic agents in treating cancer [75], there are no clinical studies on the use of miRNA modulators for preventing or treating BPD. Here, we summarize the miRNA inhibitors and miRNA mimics that have been used in preclinical animal studies for preventing BPD (Fig. 2).

Intranasal administration of miR-489, miR-34a, miR-421, miR-451 or miR-203a inhibitors during hyperoxic exposure improved alveolar development as demonstrated by increased secondary septation and radial alveolar counts, as well as reduced mean linear intercepts in rodents [25,31, 39,41,43,49]. Mechanistically, miR-34a and miR-451 inhibitors promote angiogenic activity and blood vessel maturation as well as reduce cellular senescence in the lung after neonatal hyperoxia. Hu *et al*. [40] subcutaneously injected adenovirus overexpressed-GAB1 or a miR-29a antagomir into mice prior to hyperoxia. Injection of the miR-29a antagomir further inhibited GAB1 overexpression-induced protection against alveolar simplification seen in neonatal hyperoxia. This may be due to reduced inflammatory responses and apoptosis as well as increased proliferation and angiogenesis with the mi-R29a antagomir. Intravenous injection of an miR-134–5p inhibitor ameliorated neonatal hyperoxia-induced lung injury by suppressing ferroptosis [76].

Several miRNA mimics are shown to inhibit lung injury in animal models of BPD via modulation of apoptosis, angiogenesis and inflammation. Lal *et al*. [26] administered miR-876 mimics intranasally in a single-hit (hyperoxia alone) and the double-hit (hyperoxia plus LPS) model of BPD. Injection of miR-876 mimics attenuated alveolar hypoplasia in both models. Intranasal administration of an miR-342 mimic or venous injection of miR-20b mimics significantly improved chord length and septal thickness in mice exposed to hyperoxia as neonates [54]. This may be explained by the fact that miR-342 mimic administration reduced neonatal hyperoxia-induced apoptosis and endothelial-mesenchymal transition [54]. Injection of hyperoxia exposed rats with miR-20b mimics or an miR-214 agomir alleviated lung injury, as reflected by increased number of alveoli and reduced ratio of alveolar area/pulmonary septal area [57,58]. Furthermore, miR-20b mimics suppressed hyperoxia-induced mitochondrial dysfunction by directly regulating mitochondrial dynamics. MicroRNA-214 overexpression inhibited hyperoxia-induced lung epithelial cell apoptosis via the PlGF-dependent STAT3 pathway.

It is interesting to note that above treatments with miRNA inhibitors and mimics were prophylactic. Further studies are warranted to investigate the therapeutic effects of these miRNA inhibitors and mimics on lung injury in models of BPD.

## 9. Conclusion and Perspectives

miRNAs play important roles in regulating both physiological and pathological processes, including oxidative stress, inflammatory responses, proliferation, apoptosis, senescence, and angiogenesis. All these processes contribute to the development of BPD. The dysregulation of miRNAs has been observed in the blood and tracheal aspirates of premature infants with BPD. Larger scale clinical studies are warranted to identify whether these dysregulated miRNAs are predicttors for developing BPD or biomarkers for diagnosing this disease. Preclinical studies using mice and rats suggest that manipulation of specific miRNAs could serve as potential therapeutic strategies to prevent or ameliorate lung injury in infants with BPD.

Identifying the cell-specific expression of dysregulated miRNAs in the lung of infants with BPD could be accomplished by fluorescent in situ hybridization staining. However, this technique cannot measure multiple miR-NAs in different cell types simultaneously. Single-cell microRNA sequencing, as well as spatially resolved and multiplexed miRNA quantification are cutting-edge technologies that could measure miRNAs with spatial resolution, while multiplexing directly from lung samples [77,78] could help quantify miRNA heterogeneities in tissue samples. This will lead to informed, biomarker-based diagnostics for BPD and a better understanding of the pathogenesis of this disease.

Rodents exposed to hyperoxia in the first days of life are the most commonly used models to study human BPD [79–81]. Nevertheless, these models cannot recapitulate all the characteristics of BPD. The preterm lamb models the clinical setting of preterm birth and respiratory failure requiring prolonged ventilatory support for days or weeks with oxygen-rich gas [82–84]. Perhaps, studies using larger animals will increase our understanding of the translational significance of miRNAs in the pathogenesis of BPD and allow us to use these agents in therapies for BPD.

Challenges of miRNA-based therapy include limited cellular uptake resulting in low delivery efficiency, multiple targets leading to off-target effects and toxicity [85]. Targeting miRNAs using the CRISPR/Cas system allows for precise and permanent targeting of mutations and provides an opportunity to target dysregulated miRNAs in BPD [85]. Extracellular vesicles, including exosomes and microvesicles, are considered novel tools for intercellular communication because miRNAs are packaged into them and are detectable in body fluids. miRNAs in extracellular vesicles are transferred to target cells to regulate gene expression due to their resistance to RNase digestion and high stability in the serum and various body fluids. Another promising therapeutic strategy could be to use surfactant as a delivery vehicle for miRNA mimics amd/or inhibitors in the lung since surfactant is widely used clinically.

In summary, miRNAs are often modulated in the lungs and other tissues of infants with BPD and in animal and cell models of this disease. Further defining which of the myriad of miRNAs could be targeted therapeutically will be an important goal. Innovations in detection and delivery of miRNAs will allow us to refine our approaches to mitigating BPD and its devastating consequences.

## Figures and Tables

**Table 1.** Upregulated miRNAs in samples from humans with BPD, and hyperoxia-exposed animals and cells.

	Upregulated miRNA	Samples	References

Human BPD	miR-133b, miR-7	arterial blood	[24]
	miR-34a	tracheal aspirates	[25]
	miR-1252, miR-1254, miR-130a, miR-20a, miR-20b, miR-378b, miR-876	tracheal aspirates	[26]
	miR-199a	tracheal aspirates	[27]
	miR-21	serum extracellular vesicles	[28]
	miR-628, miR-185, miR-545, miR-378	tracheal aspirates	[29]
	miR-219	tracheal aspirates, lung tissues	[30]
	miR-203a	serum	[31]
	miR-221, miR-223	plasma	[32]
	miR-184	tracheal aspirates, lung tissues	[33]

Animal models	miR-34a	mouse	[19]
	miR-21	mouse	[28]
	miR-219	mouse	[30]
	miR-203a	rat	[31]
	(pnd2) miR-20b, miR-106a, miR-128, miR-883b, miR-15b	mouse	[34]
	(pnd7) miR-122, miR-30e, miR-365		
	(pnd21) miR-133a, miR-205, miR-379, miR-449a, miR-431, let-7f		
	miR-141, miR-21, miR-34a	rat	[35]
	miR-411, miR-431, miR-699, miR-29a, miR-29c	mouse	[33]
	(pnd3) miR-490, miR-1193		
	(pnd7) miR-3584	rat	[36]
	(pnd14) miR-34c, let-7b, miR-3068, miR-872, miR-183, miR-33, miR-182, miR-322, miR-340, miR-142, miR-141, miR-96, let-7f, miR-15b, miR-449a, miR-22, miR-362, miR-301a and miR-365		
	miR-196b, miR-365, miR-146b, miR-137, miR-132	mouse	[37]
	(pnd1) miR-184, miR-347, miR-181a, miR-204, miR-132, miR-328b	rat	[38]
	(pnd3) miR-3559		
	(pnd7) miR-466b, miR-466b		
	miR-34a	mouse	[39]
	miR-29a	mouse	[40]
	miR-421	mouse	[41]
	miR-29a	mouse	[42]
	miR-451	mouse	[43]
	miR-154	mouse	[44]
	miR-125b	rat	[45]
	miR-421	mouse	[46]
	miR-194	mouse	[47]

Hyperoxia-exposed cells	miR-219	MLE 12, mouse lung primary, fibroblasts	[30]
	miR-34a, miR-34b, miR-34c	MLg	[39]
	miR-451	MLECs	[43]
	miR-421	MLE12	[46]

*Abbreviations*: miRNA, microRNAs; BPD, bronchopulmonary dysplasia; MLECs, murine lung endothelial cells; MLE12, mouse lung epithelial cells; MLg, mouse lung fibroblast cell line; pnd, postnatal day.

**Table 2.** Downregulated miRNAs in samples from humans with BPD, and hyperoxia-exposed animals and cells.

	Downregulated miRNAs	Samples	References

Human BPD	miR-152, miR-30a	arterial blood	[24]
	miR-17	plasma	[48]
	miR-489	lung tissues	[49]
	miR-29b	plasma, lung tissues	[50]
	miR-876	tracheal aspirates	[26]
	miR-30a	lung tissues	[51]
	miR-574	blood	[52]
	miR-3713, miR-3151, miR-1295, miR-1286, miR-380, miR-15a, miR-3175, miR-493, miR-3193, miR-105, miR-4300, miR-631, miR-2116, miR-4304, miR-3125, miR-4303, miR-1908, miR-205, miR-3674, miR-615, miR-4305, let-7i, miR-4330, miR-1255b, miR-125b-1, miR-24-1, miR-646	tracheal aspirates	[29]
	90 miRNAs	umbilical cord vein blood	[53]
	miR150	plasma	[32]
	miR-342	tracheal aspirates	[54]

Animal models	miR-489	mouse	[49]
	miR-876	mouse (BALF)	[26]
	miR-342	mouse	[54]
	(pnd2) miR-299, miR-139p, miR-300, miR-122		
	(pnd7) miR-335p, miR-714	mouse	[34]
	(pnd21) miR-720		
	miR-342, miR-126, miR-335, miR-150, miR-151	rat	[35]
	miR-322, miR-411, miR-431, miR-609, miR-680	mouse	[33]
	(pnd3) miR-377		
	(pnd7) miR-542, miR-99a, miR-139, miR-208a,	rat	[36]
	miR-33, miR-190a, miR-335, miR-708, miR-15b, miR-674, miR-188		
	(pnd14) miR-181c, miR-465, miR-382, miR-208a, miR-351, miR-503, miR-127, miR-664, miR-298, miR-376a, miR-186, miR-134, miR-92a, miR-378a, miR-541,miR-154		
	miR-363, miR-196a	mouse	[37]
	miR-342	mouse	[55]
	(pnd1) miR-92a, miR-6215, miR-135a, miR-449c, miR-449a, miR-376b, miR-122, miR-154, miR-543, miR-490	rat	[38]
	(pnd3) miR-338, miR-122, miR-6215, miR-1, miR-133a, miR-133b, miR-208a, miR-490, miR-741, miR-204, miR-466b, miR-466c, miR-490 (pnd14) miR-337, miR-344, miR-122, miR-1, miR-208a, miR-19b, miR-154, miR-542, miR-3559, miR-29c, miR-450a, miR-186, miR-3068, miR-29b, miR-34b, miR-500, miR-3068, miR-224, miR-201, miR-344g		
	miR-17, miR-18a, miR-19a, miR-19b, miR-20a, miR-92	mouse	[56]
	miR-20b	rat	[57]
	miR-214	rat	[58]
	miR-425	rat	[59]

Hyperoxia-exposed cells	miR-20b	AEC II	[57]
	miR-425	RLE-6TN	[59]

*Abbreviations*: AEC II, primary type II alveolar epithelia cell; BALF, bronchoalveolar lavage fluid; pnd, postnatal day; RLE-6TN, rat type II alveolar epithelial cell.

**Table 3.** Impact of dysregulated miRNAs on cellular processes in hyperoxia-exposed animals and cells.

Cell process	Change vs controls	miRNAs	Samples	References

Oxidative stress	↓	miR-425	RLE-6TN	[59]
	↑	miR-34a	mouse lung tissues	[25]
	↑	miR199a	MLECs	[27]
	↑	miR-29a	MLE12	[42]
Inflammation	↑	miR-451	mouse lung tissues	[43]
	↑	miR-421	MLE12	[46]
	↓	miR-214	rat lung tissues	[58]
	↓	miR-876	mouse lung tissues	[26]
Proliferation	↓	miR-206	A549, H441	[60]
	↓	miR-29a	MLE12	[40]
	↑	miR-206	A549, H441	[60]
	↑	miR-34a	MLE12	[25]
	↑	miR-203a	RLE-6TN	[31]
	↓	miR-342	MLE12	[54]
	↑	miR-421	mouse lung tissues, MLE12	[40,41,46]
Apoptosis	↑	miR-29a	MLE12	[42]
	↑	miR-421	mouse lung tissues	[41]
	↑	miR-194	mouse lung tissues, BEAS-2B	[47]
	↓	miR-20b	rat lung tissues, AEC II	[57]
	↓	miR-214	rat primary embryonic type II alveolar epithelial cells	[58]
	↓	miR-425	RLE-6TN	[59]
	↓	miR-21	AEC II	[65]
Senescence	↑	miR-34a	MLE12, SAEC	[66]
	↓	miR-34a	MLE12	[19]
	↑	miR-30a	HPMECs	[51]
Angiogenesis	↓	miR-200a	HUVECs	[53]
	↓	miR-203a	rat lung tissues, RLE-6TN	[31]
	↑	miR-342	mouse lung tissues	[54]
	↓	miR-451	mouse lung tissues, MLECs	[43]

*Abbreviations*: A549, Human lung adenocarcinoma epithelial cell line, metastatic cells; AEC II, rat primary type II alveolar epithelia cell; BALF, bronchoalveolar lavage fluid; BEAS-2B, human pulmonary bronchial epithelial cells; H441, Human lung adenocarcinoma epithelial cell line, nonmetastatic cells; HPMECs, neonatal human pulmonary microvascular endothelial cells; HUVECs, Human umbilical vein endothelial cells; MLE12, mouse lung epithelial cells; MLECs, murine lung endothelial cells; RLE-6TN, rat type II alveolar epithelial cell; SAEC, human small airway epithelial cells. ↑ : upregulation; ↓ : downregulation on cellular processes by miRNAs.
